# Chemical Characterization and Nutritional Markers of South African *Moringa oleifera* Seed Oils

**DOI:** 10.3390/molecules27185749

**Published:** 2022-09-06

**Authors:** Kokoette Bassey, Malebelo Mabowe, Mmamosheledi Mothibe, Bwalya A. Witika

**Affiliations:** 1 Pharmaceutical Sciences Division, School of Pharmacy, Sefako Makgatho Health Sciences University, Molotlegi Street, Ga-Rankuwa, Pretoria 0204, South Africa; 2 Division of Pharmacology, Faculty of Pharmacy, Rhodes University, Makhanda 6139, South Africa

**Keywords:** *Moringa oleifera* seeds, oils, nutritional and chemical markers, 1-dimension, 2-dimension, gas chromatography

## Abstract

*Moringa oleifera* Lam (syn. *M. ptreygosperma Gaertn*.) leaves are globally acclaimed for their nutritional content and mitigation of malnutrition. In most impoverished rural communities including Limpopo, Mpumalanga and KwaZulu Natal of South Africa, powdered leaves of *Moringa oleifera* are applied as a nutritional supplement for readily available food such as porridge for malnourished children and even breast-feeding mothers. Widely practiced and admired is also the use of the plant seed in the do-it-yourself purification of water by rural South Africans. This study aimed at identifying the chemical and nutritional marker compounds present in South African *Moringa oleifera* seed oils using high resolution 1-2-dimension gas chromatography in order to give scientific validation to its uses in cosmetics and particularly in culinary practices. Results obtained from two-dimension tandem mass spectrometry chemical signature revealed over 250 compounds, five times more than those reported from one-dimension gas chromatography. Whereas previous reports from gas chromatography-mass spectrometry analysis reported oleic acid (70–78%) as the major compound from oil samples from other countries, *M. oleifera* seed oil from South Africa is marked by *cis-*13-octadeaconic acid with 78.62% and 41.9% as the predominant monounsaturated fatty acid in the hexane and dichloromethane extracts respectively. This was followed by *cis-*vaccenic acid, an isomer of oleic acid at 51% in the acetone extract, 9-octadecanoic acid-(z)-methyl ester at 39.18%, 21.34% and 10.06% in dichloromethane, hexane and acetone extracts respectively. However, a principal component analysis with R^2^ = 0.98 of the two-dimension tandem mass spectrometry cum chemometric analysis indicated n-hexadecanoic acid, oleic acid, 9-octadecanoic acid-(z)-methyl ester and *cis*-vaccenic acid with a probability of 0.96, 0.88, 0.80 and 0.79 respectively as the marker compounds that should be used for the quality control of *moringa* seed oils from South Africa. This study demonstrates that South African *Moringa oleifera* oils contain C-18 monounsaturated fatty acids similar to oils from Egypt (76.2%), Thailand (71.6%) and Pakistan (78.5%) just to mention but a few. These fatty acids are sunflower and olive oil type-compounds and therefore place *moringa* seed oil for consideration as a cooking oil amongst its other uses.

## 1. Introduction

*Moringa oleifera* Lam is a fast-growing perennial plant, that grows up to 9 m tall, with white wood as well as a corky and gummy bark. The tree consists of brittle stem and tuberous taproots, which taste like horseradish. The tree is native to sub-Himalayan tracts of India but is mainly distributed in the Middle eastern countries of Pakistan, Bangladesh, the horn of Africa, Nigeria and South Africa in particular [1]. In addition to its other parts that are of immense benefits to man and other domestic vertebrates, *Moringa oleifera* seeds (MOSs) have found application in water purification and cosmetics formulation [2] among others. MOSs are round, have ~1 cm in diameter and are characterized by brownish semi-permeable seed hull and papery wings. The hulls of the seed are brown to black but can be white if kernels are of low viability. *Moringa oleifera* seeds grow within the pods of the *Moringa oleifera* tree. The pod can grow up to a foot in length and produce over a dozen seeds inside it. Viable seeds germinate within 2 weeks. The white wings of hulls are also present which run from top to bottom at 120 intervals. Each tree can produce around 15,000–25,000 seeds/year [1]. The seeds produce oil which can be used as alternative vegetable cooking oil as it contains many essential fatty acids such as linoleic acid, linolenic acid, behenic acid and oleic acid (Ben oil). The seeds also contain fiber, fats, minerals, proteins, vitamins B, C, and E and macro nutrients including but not limited to potassium, calcium and magnesium [3]. According to Leon et al., [1], crushed *M. oleifera* seeds in the form of a decoction found application in treating stomach pain, ulcers, poor vision, joint pain, aiding digestion as well as possessing good antimicrobial activity against numerous bacterial and fungal species. The small-to-zero side effects of phytochemicals from the seeds elicited pharmacological effects [4]. A comprehensive review by Pandey and co-workers [5] summarized the pharmacological potentials of MOS extracts to include antipyretic, profound anti-anaphylactic potential and anti-asthmatic effect from the ethanolic extract, inhibitory effect on airway inflammation by n-butanol extract and inhibition of the growth of *P. aeruginosa*, *S. aureus* and *B. subtilis* by the aqueous extract. Unpublished work in our laboratory supports the inhibitory potential of hexane, dichloromethane, acetone, methanol and water extracts of MOSs against *A. baumanni, K. pneumonia, E. coli,* and *P. aeruginosa* as well as antioxidant properties of five of the extracts. Other reported pharmacological activities such as antiplasmodial effect from the soluble lectin from the seed extract, protection from oxidative stress by the seed powder and bronchial asthma potential indicated by the finely powdered dried seed kernels of MOS have also been reported [5].

MOSs, sometimes in combination with the leaves, roots and bark, have displayed positive in vitro antimicrobial activity against *Bacillus cereus, Candida albicans, Streptococcus facalis, Staphylococcus aureus* and *Pseudomonas* aeruginosa [6], (Staphylococcus *epidermidis, Bacillus subtilis, Shigella sonnei, Escheri coli and Aspergillus* [7]. The antimicrobial activity of MOS is linked to the potent antibacterial and antifungal activity of pterygospermin, moringine and benzyl isothiocyanate which are known to help against microbial infections [3]. MOS ethanolic extract is recorded to possess anti-inflammatory properties, [6], anti-cancer properties, anti-tumor activity when tested in in-vitro [6] and thiocarbamate, and isothiocyanate are responsible for this activity in the inhibition of tumor promoter teleocidin B-4-induced Epstein–Barr virus activation in Raji cells [6]. In addition, the anti-inflammatory activity of MOS can exhibit anti-asthmatic properties, and spasmolytic activity in acetylcholine, histamine, serotonin (5HT) and barium chloride (BaCl_2_) induced bronchospasm. It has also shown protection against induced mast cells and egg albumin [6]. Diabetic mice with streptozotocin-induced diabetes fed with MOS led to a reduction in fasting blood glucose [6]. Consumption of MOS resulted in increased serum antioxidant enzymes and thus indicated that MOS can bring down reactive oxygen species (ROSs) in β-cells. Phytochemicals such as flavonoids and phenols are responsible for the antioxidant activity which helps reduce the ROSs released from the mitochondria and therefore helps in protecting the beta-cells which then control hyperglycemia [3]. Other reported bioactivities of MOS include hepatoprotective properties [6,8], diuretic properties [9], and anti-fibrotic/ulcer properties [10]. The following phytochemicals were identified and quantified from *M. oleifera* seed and seed oil from the wild provenance of Pakistan: tocopherols, sterol-24-methylenecholesterol, campesterol, campestanol, 7-campestanol, stigmasterol, clerosterol, stigmastanol, sitosterol, 7-avenasterol, 5-avenasterol, isoavenasterol and 7,14-stigmastanol, fatty acid including oleic acid, palmitic, stearic, behenic and arachidic acids [11].

This study aimed at characterizing the chemical and nutritional marker compounds present in South African *Moringa oleifera* seed oils in an attempt to promote the use of the oils in cosmetics, pharmaceuticals as well as cooking oil.

## 2. Results and Discussion

The mass obtained for each extract was 14.40 g from hexane, 2.26 g from dichloromethane, 0.96 g from acetone and 1.52 g from methanol with corresponding yields of 24.04%, 9.52%, and 6.2%, respectively. Each of the oily extracts was analyzed using one- and two-dimension gas chromatography and the chemical profiles were identified using relevant libraries.

### 2.1. One-Dimension Gas Chromatography Chemical Profile of MOS Oils

**Figure 1:**
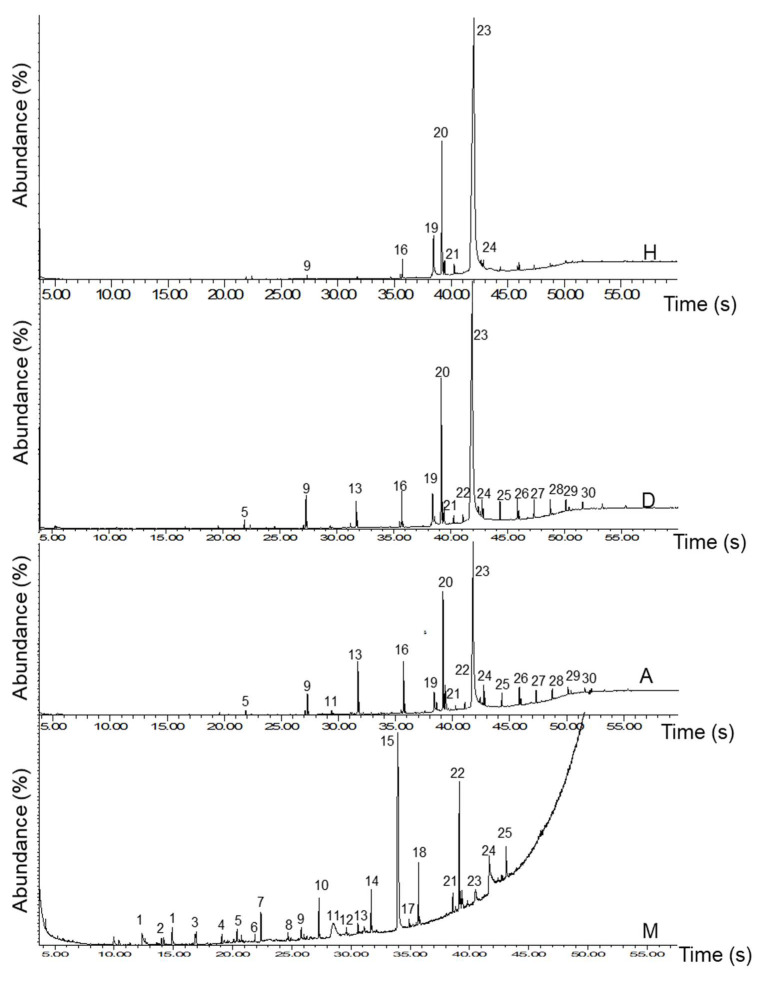
Total ion chromatogram of the MOS oil extracts. H = hexane, D = dichloromethane, A = Acetone, M = methanol.

The phytochemical profiles from the four extracts of South African *M. oleifera* are displayed in Figure 1.

A total of 106 volatiles and non-volatile compounds were detected by GC-MS. From a thorough search of National Institute Standards and Technology (NIST), Adams, and Essential Oils (EOs) mass data libraries, subsequent identification of peaks obtained from the four extracts was carried out. From the hexane extract, five compound peaks were identified while five compound peaks were also identified from the dichloromethane extract. Conversely, the acetone extracts revealed eight compound peaks and the methanol extract contained five compounds, amounting to a total of 23 identified peaks. One compound at peak number 23, *cis-*13-octadecanoic acid, is present in the hexane and dichloromethane extracts at an average of 71.19% at between 41.9–42.0 min and its isomer *cis-*vaccenic acid at 41.80 min. The co-occurrence of C_18_ fatty acid in different extracts of South African MOS oils suggests that it is of the same quality as oils from other countries that predominantly consist of these fatty acids in the same range of 71–78% [12,13,14]. To fully identify the peaks in each of the extracts, the NIST GC-MS library was searched for compounds in the library whose fingerprints could match those of the compounds detected in MOS oils extract. The results of the library search for each extract are discussed. From the hexane extract, five compounds match those of the NIST library. The peak number, retention time, peak area, percentage composition, molecular mass, fragmentation pattern and the name of each compound are summarized in Table 1.

All the compounds present in the hexane extract are of immense health benefits to man and could result in the commercialization and use of South African MOS oils in manufacturing food, food supplements and cosmetics among others. In addition to previously reported health benefits of MOS oils, this study theorizes that the use of the hexane extract of MOS oil could be beneficial in the management of viral infections including COVID-19. This is attributed to the fact that these extracts contain two unsaturated fatty acids viz., *cis*-13-octadecanoic acid (78.6%), and 9-octadeacanoic acid (Z)-methyl ester (7.9%) with recorded virucidal activities [15]. If positive results are obtained, the two unsaturated fatty acids, which are commercially available, may be tested in various combinations or alone to evaluate their virucidal activity *vis-à-vis* that of the hexane extract. From such a comparative study, one can then establish if the virucidal activity is a function of synergy between these compounds or not. 

The other secondary metabolites present in the dichloromethane, acetone and methanol were conducted, and the results are depicted in Table 1. As evident in the total ion chromatogram (TIC) of the dichloromethane, acetone and methanol extract, not all the compounds detected were identified from the library. Whereas only five were identified from the dichloromethane and methanol extracts, eight compounds were identified from the acetone extract. However, the majority of the identified compounds were fatty acids, or their methyl esters as previously reported [13]. The major compounds in the MOS oil extracted from dichloromethane, acetone and methanol are *cis-*13-octadecanoc acid with 78 and 62% composition in hexane and dichloromethane extract respectively, *cis*-vaccenic acid with 51% in acetone and 4-ethoxyphenylacetonitrile which was 15% in methanol extract. This study, therefore, proposed that any of these compounds especially the C_18_ fatty acid or its related ester may be regarded as the marker compounds for the standardization and quality control of MOS crude and commercial oils from South Africa. 

### 2.2. Two-Dimension Gas Chromatography Chemical Profile of MOS

The hexane and dichloromethane extract of South African MOS upon analysis by the GCxGC-MS yielded 282 and 129 volatiles and non-volatile compounds respectively. The biological activities of some of these compounds have been well recorded [16]. This includes interesting reviews by authors including Leone et al. [1] and Rao and co-workers [8] on the GC-MS analysis of *moringa* seeds from other parts of the world. Whereas there are 12 fatty acids and their derivatives in the dichloromethane extracts, there are more than double the number, 30, of such compounds in the hexane extract. This observation tends to support the use of hexane as the best solvent for the extraction of non-volatile compounds from South Africa *Moringa oleifere* seeds. Altogether, about 42 fatty acids and related compounds are hereby reported as present in South African MOS oils, of which n-hexadecanoic acid, oleic acid, 9-octadecanoic acid Z-methyl ester and sulfurous acid and 2-ethylhexyl isohexyl ester are the major volatile compounds in the DCM extract. 

From the hexane extract, the major fatty acids are cis-Vaccenic acid, in addition to n-hexadecanoic acid, 9-Octadecenoic acid (Z)-methyl ester, Octadecanoic acid, 13-hydroxy-, methyl ester, Benzeneacetic acid 1-methylethyl ester, Nonanoic acid, 2-phenylethyl ester, Nonanedioic acid and dibutyl ester as depicted in Table 2. The observation that was not detected in the analysis of the hexane extract but by the GCXGC-MS analysis of the same extract pinpoints the importance of the two-dimension gas chromatography.

The GCxGC-MS analysis of the MOS acetone and methanol extract separately analyzed returned *n* = 17 and *n* = 15 compounds, respectively. However, after filtering off artefacts, only the compounds depicted in Table 2 are hereby reported. These compounds were thought to be the phytochemicals that promote the medicinal use of MOS by rural South Africans [17]. Some of the compounds had selected medicinal properties based on information available in the literature on biologically active plant secondary metabolites in *Moringa oleifera* [18]. Amongst these compounds is rhodoxanthin [19] and ursane-3,16-diol, a triterpene derivative. This compound and its derivatives possess pentacyclic triterpenoids that are biologically active phytochemicals with a wide range of activities such as anti-inflammatory, hepatoprotective, anti-hypertensive, antiulcerogenic and anti-tumor [20].

The other major compounds in the methanol extract are 9-Desoxo-9-hydroxy-7-ketoingol-3,8,9,12-tetraacetate and L-Valine, N-[N,O-bis(2,4-dinitrophenyl)-L-tyrosyl]-, methyl ester. Whereas 9-Desoxo-9x-hydroxy-7-ketoingol-3, 8,9,12-tetraacetate is known to be a new phytochemical [21], L-valine is an essential amino acid classified as nonpolar. Along with leucine and isoleucine, valine is a branched-chain amino acid. Branched-chain amino acids (BCAA) are essential amino acids whose carbon structure is marked by a branch point that is critical to human life and are particularly involved in stress, energy and muscle metabolism. From the acetone extract, is 8-chloro-5-quinolinecarboxylic acid. This compound and its analogues functions medicinally as antibacterial, antifungal, antimalarial, anthelmintic, anticonvulsant, cardiotonic, anti-inflammatory, and analgesic activities [22]. Its presence in MOS once again supports the use of MOS in traditional South African medicine.

#### 2.2.1. Identification of Marker Compounds in the Non-Polar Hexane and Dichloromethane Extracts of MOS by Chemometrics

Whereas the identification of marker compounds GC-MS data is a possibility post GC-MS analysis, marker compound annotation from the GCxGC-MS data is not trivial because the instrument often generates multivariate data. To identify such marker compounds, that are essential as standards for quality control and standardization of plant, or other natural products chemometrics computation usually comes in handy.

**Figure 2:**
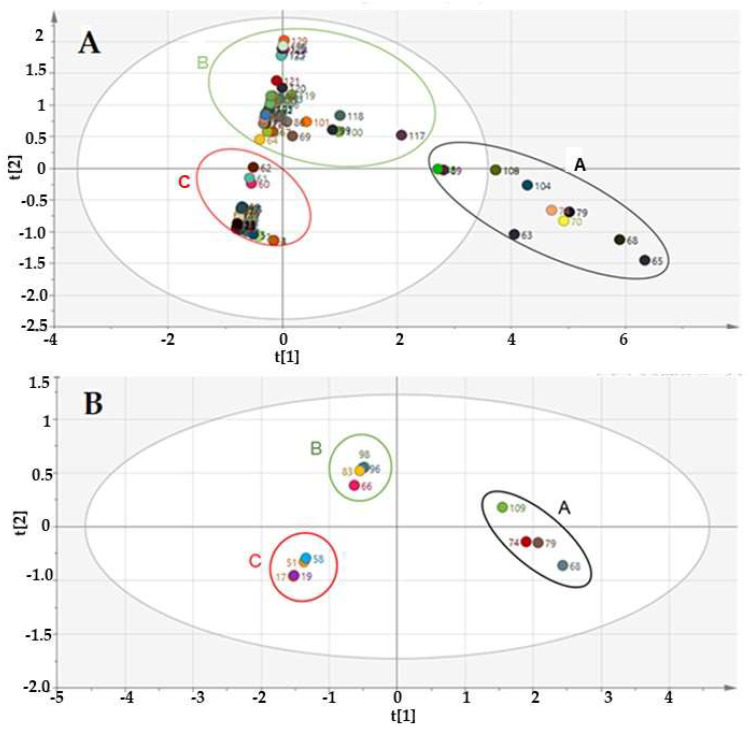
PCA score plot of (**A**) volatiles and non-volatile compounds and their clusters and (**B**) volatile compounds indicating three well-defined clusters of compounds in the hexane and dichloromethane extract of MOS oils.

The compound peaks generated from the GCxGC-TOF-MS analysis of the hexane and dichloromethane extract of the *M. oleifera* seeds were combined using the Microsoft Excel^®^ 2016 suite (Microsoft^®^ Corporation, Redmond, WA, USA). This action is a prerequisite for chemometric compatibility and analysis. All the artefacts and contaminants, such as polymeric materials and stationary phase silicate complexes were excluded from the data in Microsoft^®^ Excel before the data were made SIMCA-15^®^ compatible prior to chemometric analysis. A chemometric two-component model was constructed and used for the data analysis. An unsupervised principal component analysis (PCA) score scatter plot, depicted in Figure 2A, from the model, *R*^2^ = 0.69 ability to explain the variation or similarity in the dataset in the first component (t1) and predictive power (Q2) of 0.61 was obtained. The information from the scores plot is completely and usually independent of each other. The score plot grouped the samples into three (black or A, green or B and red or C) not very undefined clusters. However, whereas the green and red clusters are within the 95% confidence level of the analysis to suggest the intra-chemical similarity of their compounds, the compounds in the black cluster were thought to have slight inter-chemical differences from the compounds in the other two clusters.

The fact that most oils, including MOS oils, contain predominantly volatile compounds [1] necessitated the removal of the non-volatile compounds from the GCxGC-TOF-MS data set.

This was aimed at improving the clustering of the samples to draw a logical conclusion on the compound’s similarities and differences. Indeed, the new score plot (Figure 2B) exhibited an improved *R*^2^ = 077. In other words, the volatile compounds data set is better explained by the model than when the data contain non-volatiles as well. The new model grouped the volatile compounds into well-defined clusters A, B and C within the 95% confidence degree of the analysis. From the plot, Figure 2B, it is clearly observable that compounds in clusters B and C are similar because both clusters are negatively correlated. Conversely, the compounds in cluster A are positively correlated and thus signify a set of compounds that is different from those in clusters B and C. These positively correlated compounds, usually have the heaviest contribution weight (weight = p1p2 or t1) values, that is the area occupied in x-space and are often the marker compounds nominated by the chemometric model. However, a probability plot can help visualize possible marker compounds from the positively correlated cluster.

**Figure 3:**
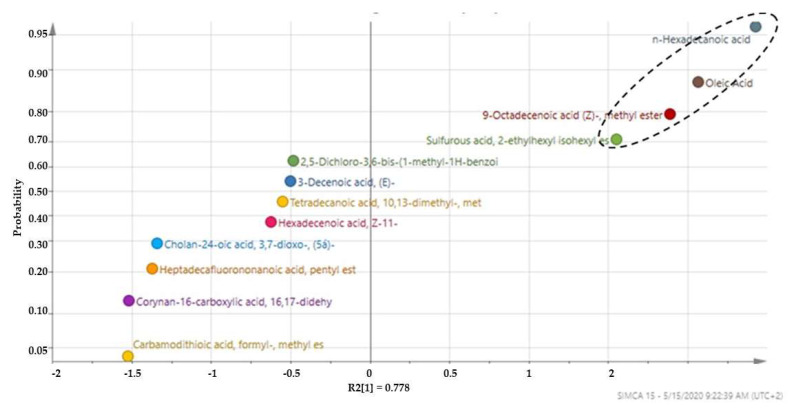
Probability plot of volatiles compounds in the dichloromethane extract of MOS oils indicating the three marker compounds insert the perforated oval.

With SIMCA-15^®^ software (Sartorius AG, Göttingen, Germany), numerous plots that are capable of nominating marker compounds in a given data set were utilized to develop a probability plot. From a probability plot, a compound with the highest probability of 0.99, which is close to 1, weighs more than the one with the least probability. As mentioned earlier, such compounds are often nominated as the marker compounds. The probability plot constructed using the volatile compounds from the dichloromethane extracts is depicted in Figure 3.

The plot revealed hexadecanoic acid, oleic acid, 9-octadecanoic (z) and methyl ester as the top three marker compounds with a probability of 0.96, 0.88 and 0.80, respectively. The weights of these compounds in the x-space were 2.43, 2.07 and 1.89, respectively as depicted in Table 3.

These three compounds are hereby reported as the marker compounds that must be present in South African MOS oils. Good quality MOS oils of South African origin must contain these compounds, otherwise the quality is compromised.

The results of this study with regards to the three marker compounds are in agreement with other authors previous reported. *M. oleifera* seed oil from Thailand reportedly contained a high level (72–74%) of oleic acid [23]. Furthermore, a review by [1] claimed that oleic acid is the predominant fatty acid, and accounts for 73.57% of the total fatty acids content of MOS oils.

**Figure 4:**
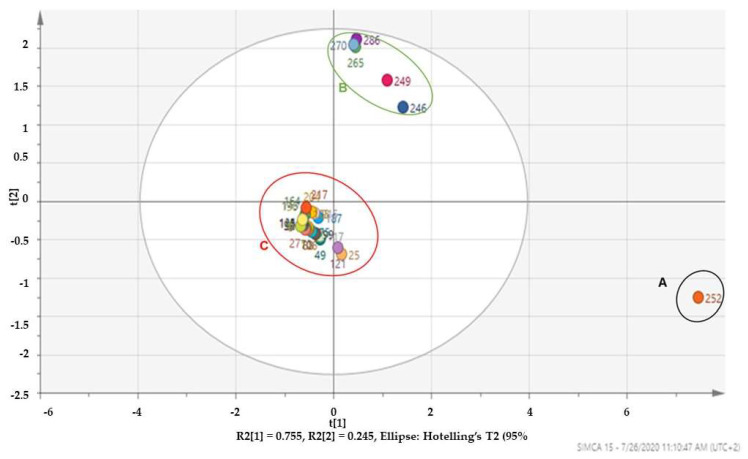
PCA score plot indicating two well-defined clusters with an outlier observation 252 of the volatile compounds in the hexane extract of MOS oils.

A separate score plot, depicted in Figure 4, with *R*^2^ = 0.75 was constructed and used to identify similarities and differences of the compounds in the hexane extract of the South African MOS oily extract. The score plot for the hexane extract depicted 3 clusters (A) with a single observation (252), that appeared as an outlier away from the Hotelling T2 95% analysis confidence level, cluster B and C. In chemometric analysis, observations that are located in the mid-point (center) of the plot usually contribute less to the clustering pattern [24]. Furthermore, as reiterated before, the positively correlated observations should weigh the most and have the highest probability of nomination as the marker compounds.

However, the observations in cluster B are positively correlated and should have higher probability of nomination as the marker compounds from the hexane extract of the MOS oils. Similar to the marker compound nomination probability approach used for the dichloromethane analysis, the hexane extracts probability plot was constructed. The plot (Figure not shown) ranked *cis-*vaccenic acid, n-hexadecanoic acid and 9-Octadecenoic acid (Z)-, methyl ester as the major marker compounds that characterized the South African MOS hexane extract. The probability values of the three compounds were 0.98% for *cis-*vaccenic acid), 0.95% for n-hexadecanoic acid) and 0.91% for 9-Octadecenoic acid (Z)-, methyl as reported in Table 4.

It is, however, not clear as to why *cis-*vaccenic acid was placed as an outlier away from the oval shape 95% confidence level of the analysis, but vaccenic acid is a structural isomer of oleic acid with the difference between both being in the position of saturation. This observation supports the nominated marker compounds of the South African MOS non-polar extracts.

In most of the studies that reported oleic acid as the predominant fatty acid in MOS oils, the analysis was conducted using a one-dimension (column) GC-MS that is characterized with compound peaks co-elution. The fact that our results from the chemometric analysis, alongside oleic acid (88%) also underscored n- hexadecanoic acid (96%) and 9-octadecanoic (z), methyl ester (80%) marker compound probability might be suggesting that, whereas these two compounds co-eluted with the oleic acid during the one dimension, GC-MS analysis, they were resolved in the second column (2D-) during the GCxGC-MS analysis. From a structural analytical perspective, one could then buttress on the observation that these compounds would have co-eluted with GC-MS analysis. Of particular interest is oleic acid and 9-octadecanoic acid (Z)-, methyl ester that differs structurally by a terminal methyl (-CH_3_) group that is possibly a product of the bio-esterification of the hydroxyl group of the oleic acid. This simply further supports that analysis of volatile compounds such as seed oils using GCxGC-TOF-MS will yield more compounds compared to the use of GC-MS.

#### 2.2.2. Identification of Marker Compounds in the Polar Acetone and Methanol Extracts of MOS

The acetone and the methanol extracts were separately analyzed by GCxGC-TOF-MS to obtain 17 and 15 non-volatile compounds, respectively. A chemometric analysis of either 17 or 15 data points is often seen as a data set that might not be statistically significant. Hence the data points were combined in Microsoft^®^ Excel and made compatible for chemometric analysis. Moreover, both solvents are polar and are expected to have extracted similar metabolites from the MOS.

**Figure 5:**
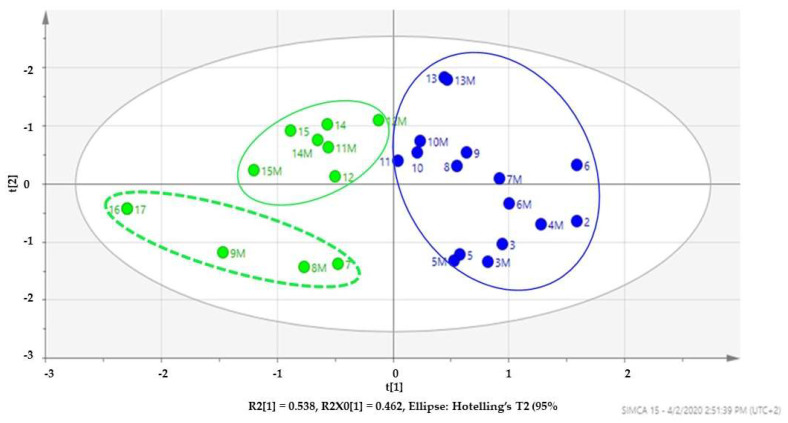
OPLS-DA Score scatter plot revealing two clusters (blue and green) of compounds present in the acetone and methanol extract. Numeric = acetone extract and alpha-numeric = methanol extract.

As previously described in Section 2.2.1, a two-component model was constructed and used to develop a scatter score plot, depicted in Figure 5, that was only able to explain about 54% variations in the data set (*R*^2^ = 0.543). Despite the R^2^ not being as close to 1, a condition for the best model for any chemometric analysis, the mid-point value (*R*^2^ = 0.5) is deemed good for any analysis. Hence the constructed model for this analysis is fit for purpose.

As evident in Figure 5, the non-volatile compounds from the acetone and methanol extract of South *M. oleifera* seed oils group into two clusters identified as the blue and the green clusters. It is worth mentioning here that the clustering was not based on the acetone nor methanol used for the extraction but was proposed as based on the similarity of the compounds within a cluster and differences between clusters. For the purposes of clarity, compounds from the methanol extract were assigned a number with letter M (e.g., 6 for compound six from acetone extract and 6M for compound 6 from methanol extract). Compounds from both extracts were present in both clusters to underpin the fact that clustering is not a function of the solvent used for extraction. Unlike the dichloromethane and hexane extracts, a PCA-X plot did not result in any pattern recognition within the data set. The construction of Orthogonal to Partial Least Square Discriminant Analysis (OPLS-DA) model score scatter plot that discriminated the data into two clusters, the blue and the green (Figure 5). However, within the green, a sub-cluster (in perforated green) highlighted on four samples with slight variation in their compounds. Hence, they are away from the other samples in the green cluster.

A contribution and probability plots nominated the marker compounds in the polar acetone-methanol oils of South African MOS. Among these compounds, 2,2′-Dipyridylamine and 29,30-Dinorgammacerane-3,22-diol, 21,21-dimethyl-, (3α, 8α, 9α, 13α, 14α, 17α, 18α, 22α) with contribution weight of t1 = 1.58 and a probability of 0.73 each were the two prominent markers compounds as depicted in Table 5. The presence of these two compounds further supports other marker compounds in the oils of MOS. That is, in addition to the fact that the oils consist predominantly of fatty acids, alkaloids, phenolics and terpene derivatives as the major secondary metabolites in the oil polar extract. A previous study highlights the importance of identifying the marker compounds present in South African *Moringa oleifera* in order to improve on the plants usage and commercialization [25]. The findings of the present study support wider usage and commercialization of MOS oils because it contains some of the fatty acids previously reported.

## 3. Materials and Methods

The South African M. oleifera seed samples were purchased from one source which is a farm that is located at Lebowakgomo in Limpopo under the trade name Patience Wellness Center (insert GPS). The plant has been verified for identity by the South African Biodiversity Institute (SANBI), Pretoria. The seeds came sealed and in brown shells.

### 3.1. Sample Preparation

The brown shell that covered each seed was peeled off by hand to afford white seeds. The white seeds were ground into fine powder using a grinding machine (Kinematica AG, Lucerne, Switzerland), mortar and pestle and later a blender. Due to the oily nature of the seeds the grinding machine had to be cleaned after five sets of seeds. Sequential Extraction of plant material using hexane, dichloromethane, methanol, acetone and water was conducted. A quantity of 223.5 g of finely powered plant material was weighed using an analytical balance. The plant material was then transferred into a 1000 mL glass beaker, 500 mL of hexane was dispensed using measuring cylinder and poured into the 1000 mL beaker with plant material. The glass beaker was then covered with parafilm. The mixture was sonicated using at a temperature of 25 °C for 30 min, after which it was left to cool for 2 min. The mixture was filtered into an Erlenmeyer flask using Whatman filter paper with a pore size of 40 then re-filtered using filter paper with a pore size of 1. This process was repeated two more times and the combined filtered was evaporated using a rotary evaporator to afford a golden oily product. The plant residue from the hexane extract was dried for 45 min and using the same protocol sequentially extracted using dichloromethane (DCM), acetone (ACN), and methanol (MeOH) to give three other oily DCM, ACN and MeOH extracts.

### 3.2. One-Dimension GC-MS

Briefly, 1.0 mg of each oily extract was dissolved in 1 mL of hexane and 1.0 µL of each extract solution was separately injected into a split/splitless mode of an Agilent 7890 GC coupled to an Agilent 5977B inert MSD mass spectrometer with a triple axis detector operating in the positive electron ionization (EI) mode, using ChemStation software (Agilent MSD ChemStation G1701EA, E.02.01.177) for the data management. The GC-MS operating conditions are given in Table 6.

### 3.3. Two-Dimension Gas Chromatography Instrument and Methodology

Solid phase microextraction of each oily extract were performed using Carboxen^®^ fibres (Sigma-Aldrich, Johannesburg, South Africa). The solvent-free prepared samples were analyzed using a LECO Pegasus 4D Time of Flight mass spectrometer (LECO Corporation, St. Joseph, MI, USA) equipped with a modified Agilent 7890A Gas Chromatograph (Agilent Technologies, Inc., Wilmington, DE, USA), a LECO GC×GC modulator and secondary oven (LECO Corporation, St. Joseph, MI, USA) and a split/splitless inlet. The column sets used were: Rxi-5 SilMS (29.5 m × 0.25 mm × 0.25 μm) as a primary column and Rxi 17 Sil MS (2.0 m × 0.25 mm × 0.25 μm) as the secondary column (Restek, Bellefonte, PA, USA). Helium was used as a carrier gas at a constant flow rate of 1 mL/min and an inlet temperature of 250 °C. An initial oven temperature of 40 °C was set and held for 0.5 min and then slowly ramped at 10 °C/min to 250 °C and then held for 0.5 min at 250 °C. The modulator and secondary oven were run at an offset temperature of 5 °C above the primary oven. The mass spectrometer was set up under the following conditions: no solvent delay because it was a SPME analysis; transfer line temperature at 250 °C; Electron ionization at −70 eV; source temperature at 250 °C; stored mass range: 45–600 μ; acquisition rate: 10 spectra/s for GC×GC-TOF-MS; detector offset voltage was set at 300 V. Retention time alignment, matched filtration, peak detection, and peak matching were done on ChromaTOF^®^ software (LECO Corporation, St. Joseph, MI, USA). Subsequent identification was done by comparison with mass spectral databases (NIST, Adams, and EO libraries). A semi quantification of each compound was calculated on the basis of peak areas and relative concentration presented in percent.

### 3.4. Chemometrics Analysis

The GCxGC-TOF-MS data were exported to Excel^®^ 2016 and made compatible for chemometrics analysis. All the artifacts and contaminants, such as polymeric materials and stationary phase silicate complexes were excluded from the data in Excel before the data were made using SIMCA-15^®^ (Sartorius AG, Göttingen, Germany) compatible prior to chemometrics analysis. Principal component analysis (PCA) and orthogonal partial least squares discriminant analysis (OPLS-DA) were performed using SIMCA-15^®^ software. From the positively correlated observations of the score plot, a column plot was constructed and analyzed, and the contribution of each compound was determined by comparing its weight against that of all the other compounds. A contribution plot for the selected compound displayed the area and the weight t1 (weight = p1p2) on the Y-axis of the plot. Compounds with the most significant weight (t1) were nominated as marker compounds by the model.

### 3.5. Statistical Analysis

The average of the replicated data obtained from the GC×GC-TOF-MS analysis was computed, and the results are presented as mean ± standard deviation.

## 4. Conclusions

The use of Moringa in South Africa has been reported for various uses. Many parts, including the seeds and its associated extracts, have been utilized.

Overall, South African MOS oils contain sunflower-type [26] and olive oil-type [27] fatty acids viz., omega-9 oleic (8.2%), stearic acid (78.6%), *cis*-vaccenic (51.0%) and palmitic acid (16.0%). Whereas sunflower and olive oils are well commercialized and edible oil, the same is not applicable to MOS oils.

From this study, we propose that MOS oils in addition to its other applications be considered and promoted as cooking oil. Furthermore, with a combined percentage of 87% per 100 mL of the oil, the in vivo virucidal activity, of the MOS hexane extract should be investigated further.

## Figures and Tables

**Table 1:** Compounds present in hexane, dichloromethane, acetone and methanol extracts of MOS oil determined by one-dimension gas chromatography.

Peak No.	Concentration %				Identified Compounds
	Hexane Extract	DCM Extract	Acetone Extract	Methanol Extract	
6	1.18				Hexadecanoic acid methyl ester
8	4.50				n-Hexadecanoic acid
9	7.91				9-Octadecanoic acid(Z)-methyl ester
23	78.62				*Cis*-13-octadecanoic acid
15	1.05				11-Eicosenoic acid, methyl ester
6		2.99			Hexadecane
14		24.71			n-hexadecanoic acid
16		14.70			9-Octadecanoic acid (Z) methyl ester
18		2.60			Methyl stearate
23		62.71			*Cis*-13-Octadecanoic acid
3			2.11		Hexadecane
5			4.71		Octadecane
8			4.43		Eicosane
10			2.51		n-Hexadecanoic acid
12			11.01		9-octadecanoic acid (Z) methyl ester
14			3.50		Docosane
17			51.60		*Cis*-vaccenic acid
20			2.16		Tetracosane
2				1.24	Hexadecane
4				15.06	4-Ethoxyphenylacetonitrile
5				1.55	Eicosane
7				3.28	*Cis*-13-octadecanoic acid, methyl ester
9				2.75	Oxazole, 2-(8)heptadecen-1-yl-4,5-dihydro

**Table 2:** Fatty acids and related esters present in Moringa oleifera seed oil dichloromethane and hexane extracts analyzed by GCXGC-MS.

Peak No.	Concentration %				Identified Compounds
	Hexane Extract	DCM Extract	Acetone Extract	Methanol Extract	
249	0.62181				9-Octadecenoic acid (Z)-methyl ester
252	5.8854				cis-Vaccenic acid
246	0.95752				n-Hexadecanoic acid
121	0.61448				Octadecanoic acid, 13-hydroxy-, methyl ester
99	0.714775				Benzeneacetic acid 1-methylethyl ester
215	0.14938				Nonanoic acid, 2-phenylethyl ester
90	0.011397				Nonanedioic acid, dibutyl ester
63		6.9588			Hexadecanoic acid
79		8.1871			Oleic acid
74		7.7338			9-Octadecenoic acid (Z)-, methyl ester
66		0.14911			Hexadecenoic acid, Z-11
96		0.066397			3-Decenoic acid, (E)-
58		0.056777			Cholan-24-oic acid, 3,7-dioxo-
83		0.009092			Tetradecanoic acid, 10,13-dimethyl-, methyl ester
2			2.8415		2,2′-Dipyridylamine
5			6.3428		Griseofulvin
7			9.0996		Rhodoxanthin
8			3.0684		17-(1,5-Dimethylhexyl)-10,13-dimethyl-3-styrylhexadecahydrocyclopenta[α]phenanthren-2-one
12			5.8482	1.24	4H-Thiopyran-4-one, tetrahydro-, 1-oxide
3			6.1133	15.06	3-[18-(3-Hydroxy-propyl)-3,3,7,12,17-pentamethyl-2,3,22,24-tetrahydro-porphin-2-yl]propan-1-ol
4			3.639	1.55	Ursane-3,16-diol, (3á,16à,18à,19à,20á)-
5			6.709	3.28	2,4,6,8,10-Tetradecapentaenoic acid, 9a-(acetyloxy)-1a,1b,4,4a,5,7a,7b,8,9,9a-decahydro-4a,7b-dihydroxy-3-(hydroxymethyl)-1,1,6,8-tetramethyl-5-oxo-1H-cyclopropa [3, 4]benz [1, 2-e]azulen-9-yl ester, [1aR-(1aà,1bá,4aá,7aà,7bà,8à,9á,9aà)]-
12			2.9016	2.75	9-desoxo-9x-hydroxy-7-ketoingol 3,8,9,12-tetraacetate
14			4.8203		L-valine, N-[N,O-bis(2,4-dinitrophenyl)-L-tyrosyl]-, methyl ester

**Table 3:** Summary of Chemometric analysis of DCM extracted MOS oil.

Possible Marker Compounds in Dichloromethane Oil of MOS		
Fatty Acid	Contribution Weight (t1)	Probability
Hexadecanoic (palmitic) acid	2.42918	0.96
Oleic Acid	2.06641	0.88
9-Octadecenoic (stearic) acid (Z)-, methyl ester	1.88866	0.80
Sulfurous acid, 2-ethylhexyl isohexyl ester	1.548	0.70

**Table 4:** Summary of Chemometric analysis of hexane extracted MOS oil.

Possible Marker Compounds in Hexane Oil of MOS		
Fatty Acid	Contribution Weight (t1)	Probability
cis-Vaccenic acid	7.45233	0.787
n-Hexadecanoic acid	1.4196	0.286
N,N′-Pentamethylenebis[s-3-aminopropyl thiosulfuric acid]	0.155692	0.214
9-Octadecenoic acid (Z)-, methyl ester	1.08897	0.407
Octadecanoic acid, 13-hydroxy-, methyl ester	0.0779159	0.184

**Table 5:** The positively correlated compounds from which the marker compounds in the acetone-methanol extracts were nominated.

Possible Marker Compounds in Acetone-Methanol Oil of MOS		
Non-Fatty Acid Compounds	Contribution Weight (t1)	Probability
2,2′-Dipyridylamine	1.58442	0.738937
29, 30-Dinorgammacerane-3,22-diol, 21, 21-dimethyl-, (3α, 8α, 9α, 13α, 14α, 17α, 18α, 22α	1.58551	0.738937
29, 30-Dinorgammacerane-3,22-diol, 21, 21-dimethyl-, (3α, 8α, 9α, 13α, 14α, 17α, 18α, 22α -	1.28101	0.738937
17-(1,5-dimethylhexyl)-10,13-dimethyl-3-styrylhexadecahydrocyclopenta[α]phenanthren-2-one	1.00519	0.738937

**Table 6:** The GC-MS conditions used for the analysis of the four MOS oil extracts.

Operation Conditions	
Injector Temperature	250 °C
Start Temperature	40 °C (hold 4 min)
End Temperature	280 °C
Ramp Rate	5 °C/min
Final hold time	8 min
Total Time	60 min
Carrier Gas	Helium
Carrier Gas Flow	0.9 mL/min
Column Parameters	5% phenyl, 95% methyl siloxane, 30 m × 0.25 mm × 0.25 µm
Solvent Delay	3.50 min
MS Source Temperature	230 °C
MS Quad Temperature	150 °C
Electron Energy	70 eV
Scan Range	40–550 amu

## Data Availability

The data used in the current study are contained within the article.

## Sample Availability

Samples of the compounds are not available from the authors.
